# Routine immunization against *Streptococcus pneumoniae* and *Haemophilus influenzae* type B and antibiotic consumption in India: a dynamic modeling analysis

**DOI:** 10.1016/j.lansea.2024.100498

**Published:** 2024-10-16

**Authors:** Chirag K. Kumar, Alec C. Gleason, Giridara Gopal Parameswaran, Amit Summan, Eili Klein, Ramanan Laxminarayan, Arindam Nandi

**Affiliations:** a Princeton University, Princeton, NJ, USA; b One Health Trust, Bengaluru, India; c One Health Trust, Washington, DC, USA; d Department of Emergency Medicine, Johns Hopkins University, Baltimore, MD, USA; e Population Council, New York, NY, USA

**Keywords:** Vaccinations, Antibiotic use, *Streptococcus pneumoniae*, *Haemophilus influenzae* type b, PCV, Hib, Antimicrobial resistance, LMICs, Agent-based modeling

## Abstract

**Background:**

Childhood vaccinations can reduce disease burden and associated antibiotic use, in turn reducing the risk of antimicrobial resistance (AMR). We retrospectively estimated the population-level reductions in antibiotic use in India following the introduction of vaccines against *Streptococcus pneumoniae* and *Haemophilius influenzae* type B in the national immunization program for children in the mid-2010s and projected future gains to 2028 if vaccination coverage were to be increased.

**Methods:**

Using IndiaSim, a dynamic agent-based microsimulation model (ABM) for India, we simulated the spread of *Streptococcus pneumoniae* and *Haemophilius influenzae* type B (Hib) among children to estimate reductions in antibiotic use under the scenarios of: (i) pneumococcal and Hib vaccine coverage levels equivalent to the national coverage of pentavalent diphtheria-pertussis-tetanus third dose (DPT3) compared to a baseline of no vaccination, and (ii) near-universal (90%) coverage of the vaccines compared to pre-COVID national DPT3-level coverage. Model parameters, including national DPT3 coverage rates, were based on data from the National Family Household Survey 2015–2016 and other published sources. We quantified reductions in antibiotic consumption nationally and by state and wealth quintiles.

**Findings:**

We estimate that coverage of *S. pneumoniae* and Hib vaccines at the same level as DPT3 in India would translate to a 61.4% [95% UI: 43.8–69.5] reduction in attributable antibiotic use compared to a baseline of zero vaccination coverage. Increases in childhood vaccination coverage between 2004 and 2016 have likely reduced attributable antibiotic demand by as much as 93.4% among the poorest quintile. Increasing vaccination coverage by an additional 11 percentage points from 2016 levels results in mortality and antibiotic use across wealth quintiles becoming increasingly similar (p < 0.05), reducing in health inquities. We project that near-universal vaccine coverage would further reduce inequities in antibiotic demand and may eliminate of outbreak-associated antibiotic use from *S. pneumoniae* and Hib.

**Interpretation:**

Though vaccination has a complex relationship with antibiotic use because both are modulated by socioeconomic factors, increasing vaccinations for *S. pneumoniae* and Hib may have a significant impact on reducing antibiotic use and improving health outcomes among the poorest individuals.

**Funding:**

The 10.13039/100000865Bill & Melinda Gates Foundation (grant numbers OPP1158136 and OPP1190803).

**Research in context**

**Evidence before this study**

The direct public health and economic benefits of childhood vaccines have been well-quantified and have indicated that childhood vaccines are highly cost-effective. Recent evidence has emerged that vaccines could reduce antibiotic use and the risk of antimicrobial resistance, a threat to global childhood survival targets. India experiences a particular nexus of these challenges—resource constraints mean the population is particularly prone to the difficulties of controlling antimicrobial resistance and a high birth rate motivates continued strategies to ensure childhood well-being. India has made significant progress in implementing widespread childhood immunizations—such as those against *Streptococcus pneumoniae* and *Haemophilius influenzae* type B—as part of their universal immunization program, which has significantly reduced childhood mortality. By averting illness, such vaccines have likely also reduced antimicrobial use, but the dynamic relationship between vaccine coverage, health outcomes, and antibiotic use has not been previously examined. Insights into cases and antibiotic use averted at the state and wealth quintile level are valuable for evaluating existing vaccination programs and informing future efforts.

**Added value of this study**

To better understand how vaccination coverage may impact antimicrobial use in resource-limited settings, we expanded a dynamic individual-based model of children in India to explore the relationship between *Streptococcus pneumoniae* and *Haemophilius influenzae* type B transmission, vaccination, and corresponding disease-associated health outcomes and antimicrobial use by state and wealth quintile. Using data from the fourth and fifth rounds of Indian's National Family Household Survey (NFHS), we estimated that retrospective vaccination efforts have reduced antimicrobial use from *S. pneumoniae* and *H. influenzae* type B from 7% to less than 4% of India's total consumption. We estimate that increasing vaccination coverage could reduce disproportionate antibiotic demand across wealth quintiles.

**Implications of all the available evidence**

Excess antimicrobial use and antimicrobial resistance is a growing threat for India. Our findings indicate that existing vaccination efforts have been successful in reducing antimicrobial use due to *S. pneumoniae* and *H. influenzae* type B. However, continued vaccination may have significant benefits in further reducing antimicrobial use and health and antimicrobial demand inequities across Indian states. Efforts to ensure increases in vaccination levels could assist antimicrobial stewardship efforts.

## Introduction

Vaccines can help reduce the burden of bacterial and viral infections.^1^ In low- and middle-income countries (LMICs), which have historically higher infectious disease burden than the rest of the world, childhood vaccinations have potentially averted 120 million deaths among those born from 2000 to 2030.^2^ Vaccines can also help mitigate antimicrobial resistance (AMR)—a leading global health challenge^3^—by preventing infections that would have otherwise been treated with antibiotics.^4^, ^5^, ^6^, ^7^, ^8^, ^9^, ^10^, ^11^ Although antibiotic use and the risk of AMR varies widely across LMICs, AMR poses an additionally noteworthy challenge in LMICs where many factors including antibiotic misuse and poor sanitation hinder antibiotic stewardship efforts and promote the emergence of novel antimicrobial resistance genes. In India, numerous novel antimicrobial resistance genes have emerged, often first identified in children and causing a disproportionate rate of illness among children that are of significant global health concern.^12^, ^13^, ^14^, ^15^

Despite an emerging body of evidence on the value of vaccines and their importance in mitigating antimicrobial resistance especially in LMICs,^16^^,^^17^ there is limited work that aims to mechanistically understand how vaccination impacts antibiotic use. A recent meta-analysis of 96 studies of vaccinations and antibiotic use worldwide found that PCV vaccines may have reduced antibiotic use among children, though the underlying evidence base was limited; there were no conclusive data for Hib vaccination on reducing antibiotic use.^9^ Although various studies have shown that PCV and Hib vaccines may have reduced antibiotic use among children,^16^^,^^18^ they have primarily evaluated the relationship between vaccinations and antibiotic use at the individual or ecological level using household surveys or aggregate data rather than mechanistic modeling.^10^^,^^11^ Such analysis may not account for disease transmission dynamics which has important implications for the health burden of the disease, associated antibiotic use and resistance, vaccine-derived and secondary immunity protection, and vaccination policy at the community level. Moreover, antibiotic access is greatest among the wealthiest individuals,^19^, ^20^, ^21^ but vaccination programs have aimed to be equitable^22^; thus, the benefit of vaccines, particularly for the poorest quintiles, is poorly quantified and may be undervalued.

In recent years, India has introduced vaccinations against bacterial pathogens *Streptococcus pneumoniae* and *Haemophilius influenzae* type B (Hib) which are common causes of pneumonia and other serious disease manifestations in children.^23^ In 2015, the most recent year for which data are available, there were an estimated 1.84 million cases and 84,000 deaths from pneumococcal and Hib pneumonia among children under the age of five in India.^24^ However, the authors of these estimates acknowledge that the disease burden could be an underestimate and their sensitivity analyses suggest that the number of deaths could be up to 50% greater. In 2017, a pneumococcal conjugate vaccine (PCV tri-decavalent or PCV13) that confers immunity against commonly-circulating serotypes of *S. pneumoniae* was introduced through the national childhood immunization program (known as the Universal Immunization Programme or UIP) in high-burden states and its availability was gradually expanded to all states by 2021, with coverage among target children reaching 75% by 2022.^25^ Hib vaccination was introduced as part of the pentavalent vaccine—diphtheria, pertussis, and tetanus (DPT), hepatitis B, and Hib—in the states of Tamil Nadu and Kerala in 2011 and its availability was expanded nationally by 2017. India achieved 91% coverage of the DPT third dose (DPT3) among children under the age of two years by 2019, before experiencing some reductions in coverage due to health system challenges induced by the COVID-19 pandemic.^26^, ^27^, ^28^

In this study, we expanded a representative, dynamic, agent-based model (ABM) of children in India that simulates *S. pneumoniae* and Hib transmission at the district level and examined the impact of vaccinations against *S. pneumoniae* (PCV13) and Hib (Hib-containing-pentavalent) on antibiotic use by wealth quintile. We projected the retrospective reductions in antibiotic use from 2004 when such vaccines were first made available to 2016 when the latest data prior to COVID-19 is available for vaccine coverage as a status quo scenario compared with a baseline of no vaccinations. We then projected the potential reductions in future antibiotic use if vaccine coverage were to be increased from 2016 levels to near universal (90%) vaccine coverage by 2028. We examined how antibiotic use changes differ by geography and wealth quintile across both scenarios.

## Methods

We expanded a representative agent-based model (ABM) of children in the Indian population with resolution to the district level (Supplementary Fig. S1). This model is an update of IndiaSim, a previously validated ABM that was used to evaluate the benefits of health interventions in India such as home-based neonatal care, water and sanitation, treatment for epilepsy, and childhood vaccines.^29^, ^30^, ^31^, ^32^, ^33^, ^34^ Individuals in the model interact along a small world network, with children (ages zero through 14; i.e., under the age of fifteen) within a district being fully connected (i.e., all children in a district are connected with an interaction probability given from a contact matrix) and children between districts experiencing quasi-independence. This meant children in two separate districts did not directly interact or transmit disease but could still be impacted by each other: in particular, susceptible children randomly acquired an infection at a probability proportional to the incidence of the disease in surrounding districts. This served to model the complex interactions (ex., a child's parents traveling between two districts and being a carrier for a disease that then afflicted a child) that would be near-impossible to explicitly model.

Three types of data were required for this model: (a) epidemiological parameters, (b) population structure, and (c) person-to-person interactions relevant to *S. pneumoniae* and Hib transmission. All model parameters relevant to (a) disease transmission (such as incubation periods, symptom duration, time until antibiotics become effective, probabilities of transmission, cross-protectivity of infection by a previous serotype) were drawn from previously published papers and cohort studies that took epidemiological and ecological approaches to understand *S. pneumoniae*, Hib, and their respective vaccines. See Appendix Table 1 for a list of full model parameters. (b) Demographic variables were gathered from the National Family Household Survey 2015–2016 (NFHS-4) as it was directly comparable to the last Indian population census. Health variables were gathered from NFHS-5 which took place from 2019 to 2021 to most accurately reflect outcomes among Indian children. Population structure was determined from the National Family Household Survey 2015–2016 (NFHS-4) conducted in India and modeled to be representative of the data gathered in this survey: this survey collected data on 265,653 children under the age of 3 years and provides district-level data on a variety of factors including demography and variables such as family structure, wealth level, vaccination status, and location.^35^ NFHS-4 was part of the standardized Demographic and Health Surveys (DHS) that are conducted in over 90 countries; NFHS-4 covered 601,509 households and 2.87 million individuals across all states and union territories of India. Although NFHS-5 data contains demographic data, at the time of modeling, it was not comparable to the latest Indian census. Only NFHS-4 data was fully comparable to the latest Indian census, but NFHS-5 data was still used for health outcomes as it is most up-to-date.^36^ Appendix Table 1 describes what parameters were from NFHS-4 or NFHS-5. (c) Finally, given emerging evidence that points to the importance of heterogenous interactions (i.e., so-called “superspreading”) and distinct social contact patterns in modeling disease spread and vaccinations, we aimed to properly account for social contact heterogeneity that may impact *S. pneumoniae* and Hib transmission. We used child interaction contact matrices that described the probability that two children interact with each other based on their ages and the setting (e.g., school, community, or home) by Indian state. The contact matrices were generated from previous modeling studies that used these DHS data to evaluate the likely contacts of individuals and were calibrated to real-world cohort interaction studies that have shown promise in predicting disease spread.^37^

We adapted existing transmission models of *S. pneumoniae* and Hib for implementation in this ABM (described in detail below). Broadly, individuals in all potential disease statuses caused by either *S. pneumoniae* and Hib were included: individuals were either not colonized and healthy, colonized and healthy, colonized and symptomatically infected, or dead. Transmission could occur between individuals who were colonized and those who were not, depending on the time since initial infection, transmissivity of the serotype, and whether the person who had been in contact with the infectious agent had immunity to the serotype. Individuals were vaccinated with a probability dependent on their state of residence and wealth quintile, determined from NFHS-4 data. Likewise, if a child were experiencing symptoms from either *S. pneumoniae* or Hib, they could seek treatment depending on their wealth quintile. Retrospective simulations—starting in 2004 and ending in 2015—were run with a time step of one day for 1 year as a calibration period until a steady state/equilibration of disease carriers and immunity was reached; simulations that were run from the present day going forward were initialized to start from the last day of the previous simulations, inheriting the circulating serotypes and therefore not requiring an additional burn-in/equilibration period. Additional model details and a table of all parameters are available in Supplementary Appendix 1.

### Demographic model

Transmission dynamics depend on the underlying population demography and social network. Compared to traditional compartmental models, ABMs allow for easily incorporating social networks when such data are available. In IndiaSIM, we modeled the social interactions of 700,000 representative children under the age of fifteen (i.e., those age groups contributing to transmission) in all 707 Indian districts of the country. Since the model was run for multiple years, we included vital dynamics: new children were introduced into the simulation, and children were no longer considered once they were older than fifteen. Transmission, symptoms, and antibiotic use were modeled as a function of age (Appendix Table 1). Birth rates are calculated by district based on NFHS-4 data and new children are continuously introduced into each district at birth. New children are representatively assigned a wealth quintile and vaccination status. As such, our model is a representative population simulator of children in India.

### Disease dynamics

We independently modeled infection from *S. pneumoniae* and Hib in children; however, we calibrated our model to cumulative cases from both diseases, and we report the incidence and averted antibiotic use from both diseases together. This enabled us to plot our infection curves together and in terms of the number of individuals presenting with pneumonia-like illness, a useful measure for health systems that are trying to estimate burden. Although our model considers children across all possible disease states following colonization, children who are colonized but not presenting with symptoms would not receive antibiotic treatment, so plotting them in our incidence counts may be misleading. Instead, we extracted, analyzed, and plotted only individuals who had pneumonia-like illness, with symptom incidences determined from the literature (Appendix Table 1). Once colonized, children could be either infected and symptomatic or asymptomatic for a period of time (parameters in Appendix Table 1).

We employed a pre-assignment process for infection. At the start of a child's infection, we assigned an individual reproductive number that is drawn from a negative binomial distribution with mean equal to the disease's overall reproductive number (based on cohort studies; Appendix Table 1). We distributed those infections over the agent's infectious period, which followed a skewed triangle distribution; this approach is consistent with previous modeling studies^38^^,^^39^ and work that recreates transmission models from cohort studies.^40^, ^41^, ^42^ The length of infection was drawn from cohort studies, and following colonization, children could either remain healthy and still transmitting at a lower rate or presenting with disease symptoms and transmitting at a higher rate (Appendix Table 1).

The above paragraph describes the overall infection schema: however, *S. pneumoniae* has many circulating serotypes that have different epidemiological dynamics. Rather than explicitly modeling each of these serotypes, we introduced individual variability to account for different serotypes modeled implicitly—this included modeling all serotypes, even those that are not targeted by vaccination, and modeling that vaccination would have varying efficacy against each serotype. Individual variability and changes to overall dynamics were based on previous work by Cobey and Lipsitch,^43^ which was also used to estimate the impact of infection by a serotype on future colonization both potentially by the same and different serotypes. In other words, rather than tracking and modeling the transmission of each serotype, infected individuals have transmission probabilities and cross immunity protectivity drawn from an initial population-level distribution that is aggregated over all circulating serotypes. The prevalence of a given serotype changes as the model iterates through time to reflect the serotypes that are circulating which may change with vaccination, enabling implicitly modeling the serotype replacement effect.

For both diseases, transmission between individuals could occur through contact with a carrier or symptomatic individual. We did not explicitly model serotype replacement effects because we did not explicitly model the prevalence of each serotype: however, we enabled our analysis to consider serotype replacement effects by (a) modeling differential efficacy of the vaccine by serotype and that serotype prevalence changes over time and (b) plotting antibiotic use from vaccine-sensitive cases when analyzing the impact of additional vaccination that would surpass the coverage required for herd immunity. By analyzing vaccine-sensitive outbreak-related cases, we are limiting our analysis to focus on those cases that could be averted by vaccination, rather than attempting to predict the exact emergence of a particular serotype that vaccines do not target many years out into the future.

Individuals were vaccinated against the disease at rates consistent with NFHS data for both diseases (discussed in detail later). In turn, each vaccine had a given efficacy (value taken from cohort epidemiological studies of the two diseases), which impacted individual disease transmission. For *S. pneumoniae,* transmission was also modulated by seasonality, with changes in the transmission rate drawn from studies linking climate to the spread of *S. pneumoniae.*^44^ Besides person-to-person transmission, we included community transmission at a rate proportional to the existing prevalence of the disease within a state and the average transmission rate across all children within that state.

We assumed that the rate at which an infected agent seeks antibiotics following the onset of symptoms depended on their wealth-quintile, as drawn from cohort studies (Appendix Table 1). Any changes to these values only change the relative ranking of each wealth quintile in their total antibiotic use. Based on existing rates of antibiotic resistance in *S. pneumoniae* and Hib,^45^, ^46^, ^47^ we also modeled whether the antibiotic would be effective. If it was not, the agent may again seek antibiotics based on wealth quintile and symptomatic progression. If the individual is unable to clear the disease through treatment or does not seek antibiotics, they die at a rate based on reported deaths by age. The remaining children recovered without any treatment needed, but a fraction of them still presented with symptoms. All model parameters and their values are provided in Appendix Table 1.

### Calibration and validation

Agent-based models must be calibrated to and be able to replicate historic disease incidence to provide trustworthy forward projections. In our modeling approach, two key parameters needed to be calibrated to replicate historical data: colonization rates and individual-level variability that acted as a proxy for implicitly modeling various circulating serotypes of *S. pneumoniae*. We used available data on *S. pneumoniae* and Hib disease incidence and mortality in 2015 from various sources and by Indian state (Appendix Table 1) as the output we aimed for our model to reproduce after calibration of the unknown parameters.

To address these data-based short-comings, we fit our model to minimize the deviation between predicted and actual mortality (prioritized) and incidence from *S. pneumoniae* and Hib in 2015. Via this approach, we fit colonization rates (using initial conditions from source data specified in Appendix Table 1 as the starting point for our fitting algorithm) and individual-level variability among children in 2004. We used these parameter fits to initialize our model and then run it for the equilibration period (discussed below); no further calibration was needed as all additional variables were computed internally by the model. The fully calibrated model was used to simulate health and antibiotic outcomes. Note that no additional calibration was needed when running prospective simulations because the two scenarios covered consecutive time periods: the prospective simulations were initialized from the last step of the retrospective simulation.

### Vaccination scenarios and sensitivity analyses

We consider two vaccination scenarios: (i) vaccination rates increasing linearly from zero to district-specific DPT3 coverage level in NFHS-4 data (i.e., retroactively modeling the impact of vaccination and comparing to no vaccinations) from 2004 to 2016 and (ii) vaccination rates increasing linearly from estimated rates of DPT3 vaccination from NFHS-4 to 90% (i.e., near universal immunization coverage and comparing to existing vaccination levels) from 2016 to 2028. We modeled children as either being fully vaccinated or not vaccinated at rates equivalent to the district level vaccine coverage from the NFHS data; partial vaccination of children was not modeled as such data was not available. We modeled PCV13 vaccination as occuring at either two, four, six, or twelve months of age (in line with India following a three plus one PCV13 immunization schedule), and Hib-containing-pentavalent vaccination ocurring at two months of age and finished by fifteen months of age.

Because actual PCV13 vaccine coverage data have yet to be available at the district level in India, when modeling the retrospective impact of vaccinations, our model for *S. pneumoniae* assumed that coverage rates were equivalent to DPT3 coverage. This is because both pneumococcal and DPT3/Hib-containing-pentavalent) vaccines are given within 14 weeks since birth, and it is likely that their coverage rates would be similar to DPT3. Likewise, we report statistics from the two diseases collectively as they hinge upon the same vaccinations. Finally, since exact data on vaccine distribution by year was unavailable (i.e., vaccine coverage in a given district by year), we assumed a linear increase in vaccine coverage, following the canonical modeling maxim of introducing as few assumptions as possible that could adversely impact interpretation of results.

For both scenarios, we considered health outcomes and antibiotic use changes by wealth quintile. To facilitate visualization, we plot symptomatic cases that are most likely to be impacted by vaccination, based on meta-estimates of the prevalence of various degrees of severity of invasive pneumonia.^48^^,^^49^ We also conducted a variety of sensitivity analyses and accounted for uncertainty in all parameters in our model. The model is run for one year as a calibration equilibration period to establish carriage rates and base levels of immunity in the population. We ran each model for 12 years starting in 2004 and ending in 2016. This includes initial vaccination efforts prior to the commencement of the Indian Universal Immunization Program—Mission Indradhanush, which served as an intensified national vaccination campaign—in 2014 in our model. We ran each model for ten years in the future when projecting the impact of increased vaccination. Each model was run 200 times (i.e., independent replicates), and we present the 50th, 2.5th, and 97.5th percentiles (i.e., the “credible intervals”) over the 200 independent replicates as summary statistics. Unless otherwise mentioned, to test for statistically significant difference in medians between two distributions, we use a two-sided paired Mood's Median test. The model was developed in Julia v1.7.3 using the Agents.jl package.

### Role of the funding source

The funders of this study had no role in study design, data collection, data analysis, interpretation, or writing of the report.

## Results

### Vaccine-averted illness and antibiotic use

**Fig. 1:**
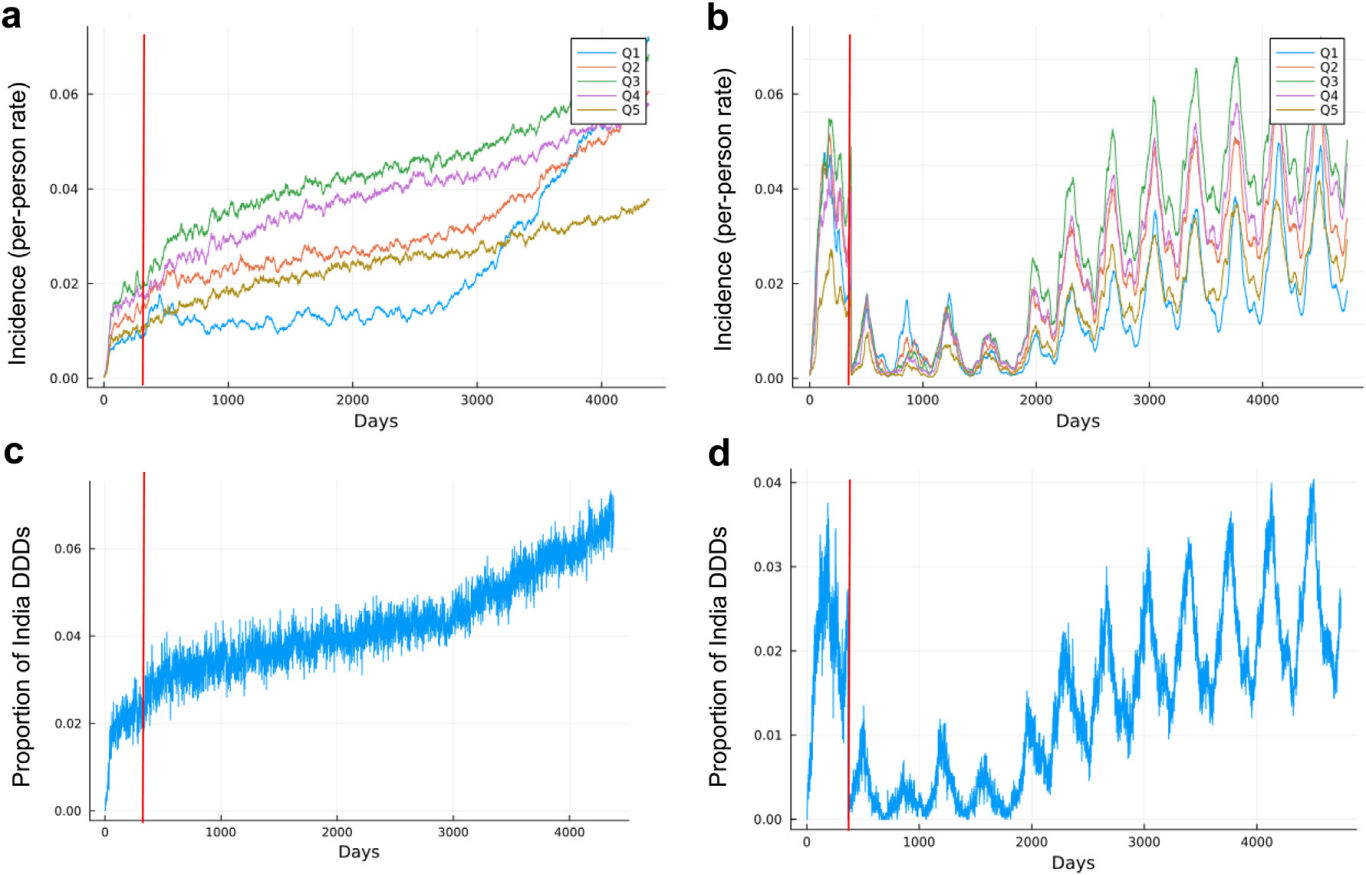
PCV13 and Hib-containing-pentavalent vaccination have averted antibiotic use. Fully calibrated whole-India agent-based model run from 2004 to 2016 to determine health and antibiotic use impacts of PCV13 and Hib-containing-pentavalent vaccination. Two scenarios are compared: no vaccinations (left), and vaccinations as they increased from first implementation in 2004 to integration into universal immunization program by 2016 (right). Symptomatic infection curves showing per-person incidence rate (i.e., the rate that a given child may become sick by *S. pneumoniae* or Hib) by wealth quintile for a scenario with no vaccinations (a) and increasing vaccinations from zero to 2016 coverage levels (b) by wealth quintile. Proportion of total daily defined doses (DDDs) of antibiotics in India used for *S. pneumoniae* and Hib for a scenario with no vaccinations (c) and increasing vaccinations to 2016 coverage levels (d). The red line marks the point at which the modeled equilibration period ends.

We estimated the impact of the pre-COVID levels of PCV13 and Hib-containing-pentavalent vaccination on averting childhood pneumonia illness and associated antibiotic use by comparing to a counterfactual scenario of there being no vaccination. Fig. 1 shows median projected symptomatic (i.e., cases where symptoms would be averted from vaccination) infection curves and antibiotic use if there were to be no vaccination (1A; 1C) and if vaccines were distributed over the last 12 years until 2016 coverage levels were reached (1B; 1D). By reducing the prevalence of *S. pneumoniae* and Hib, vaccination would significantly reduce associated antibiotic use. We projected that providing PCV13 and Hib-containing-pentavalent vaccines at current levels of other vaccines in UIP would decrease yearly pneumonia-like illness by 36.8% [22.3–67.9] and associated antibiotic use by 61.4% [43.8–69.5]. In the scenario with no vaccination, we estimated that vaccine-avertable pneumonia-like illness incidence would approach a near constant value of 3.5 cases per 100 children until day 3000 (Fig. 1a). Comparatively, with vaccination, incidence ultimately decreased and reflected the seasonal pattern of transmission (peaks in the summer months) of *S. pneumoniae* used to parameterize the model (Fig. 1b). The cycles in disease burden reflect the seasonal pattern of *S. pneumoniae* transmission that was used to parameterize the model: the reduced burden from vaccination enables these trends to be observed, though the cyclical trends were not a function of vaccination.

Peak daily antibiotic use would plateau with vaccination as opposed to a consistent increase without vaccines. Antibiotic use from *S. pneumoniae* and Hib with vaccination is projected to not exceed 4% of India's total daily defined doses (DDDs) (Fig. 1d). Without vaccination, antibiotic use would exceed 7% of total DDDs and continues to grow (Fig. 1c). Despite wealthier individuals having greater access to antibiotics (Appendix Table 1), Fig. 1a shows that the growth in disease incidence in poorer quintiles (Q1 and Q2) is associated with such increases in antibiotic use. Following the equilibration period, disease incidence is highest among wealthier populations (Q3 and Q4), and Q1 and Q5 have the lowest disease incidence, though differences between wealth quintiles remain minimal (overlap of 95th percentile credible intervals ranging from 9% to 17%). However, after day 2900, incidence in Q1 would rise rapidly and remains increasing, so that the greatest incidence is observed in Q1. A similar pattern is observed in Q2, though the growth in incidence is less (34.6% [27.5–39.8]).

Initially, the highest disease incidence is observed in Q3 with Q1 and Q5 having the lowest incidence on average, indicating a complex relationship between wealth quintile and disease incidence (discussed in detail below). However, Q3 having the greatest incidence is consistent with observed dynamics of vaccines being available among the poorest (Supplementary Fig. S2 shows that efforts have been made to get the poorest states access to vaccines) while antibiotics are most readily accessible to the wealthiest (Appendix Table 1), meaning that the middle-class has relatively lesser availability of both. When vaccinations are present, for the first five years, there would be no statistical differences between wealth quintiles (p = 0.27). Thus, we estimate that vaccination reduced health inequities by delaying the stratification of health outcomes by wealth quintile.

### Vaccinations reduce inequities in antibiotic use

**Fig. 2:**
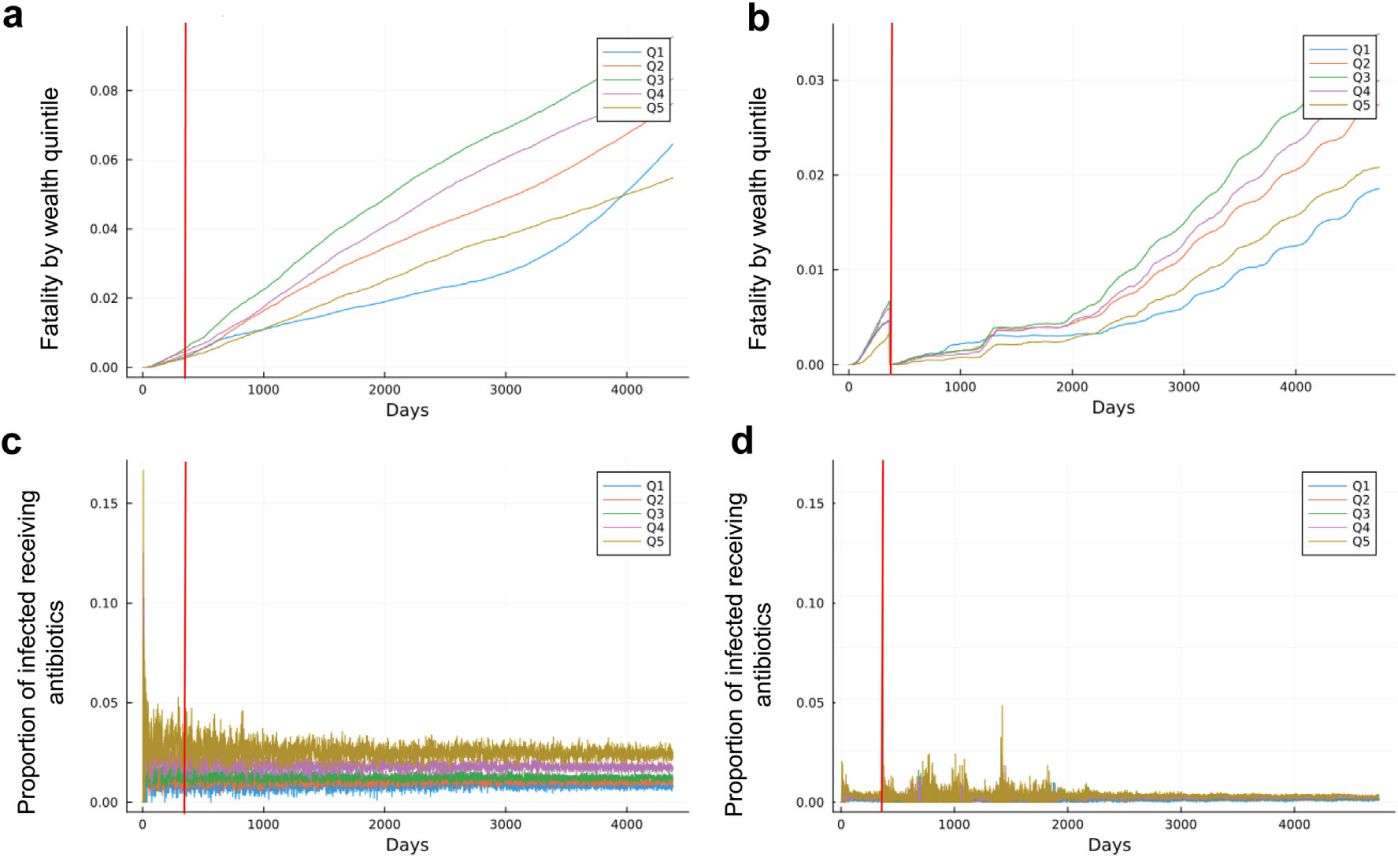
PCV13 and Hib-containing-pentavalent vaccination have improved health equity and equitable distribution of antibiotics. Fully calibrated whole-India agent-based model run from 2004 to 2016 to determine health and antibiotic use impacts of PCV13 and Hib-containing-pentavalent vaccination. Two scenarios are compared: no vaccinations (left), and vaccinations as they increased from first implementation in 2004 to integration into universal immunization program by 2016 (right). Cumulative mortality curves (total proportion of wealth quintile that has succumbed to *S. pneumoniae* or Hib) by wealth quintile for a scenario with no vaccinations (a) and increasing vaccinations to 2016 coverage levels (b) by wealth quintile. Proportion of infected agents receiving antibiotics for *S. pneumoniae* and Hib for a scenario with no vaccinations (c) and increasing vaccinations to 2016 coverage levels (d). The red line marks the point at which the equilibration period ends.

Fig. 2 presents an analysis of how vaccination would impact inequities in antibiotic use across wealth quintiles. Fig. 2a and c show cumulative mortality and antibiotic use by wealth quintile were there to be no vaccines, and Fig. 2b and d show the same for a scenario with vaccines that increase to reach 2016 levels (Supplementary Fig. S1), mirroring real-world progress in India's UIP. Fig. 2a and b underscore the dynamics in the infection curves in Fig. 1a and b. Fig. 2c and d underscore that antibiotic use would decrease with vaccination; that decrease is evident across all wealth quintiles, ranging from a 93.4% reduction for Q5 to a 33.6% reduction for Q1. When vaccines were not available, antibiotic usage would remain at nearly constant proportions for each wealth quintile throughout the entirety of the simulation (2.0% [1.6–2.3] for Q5 to 0.5% [0.3–0.6] for Q1). Reflecting differential access to treatment, antibiotic usage was highest and responded to changes in incidence (quantified by the highest correlation between incidence and changes in antibiotic use of R^2^ = 78%) among the wealthiest (Q5) while it was lowest and the constant among the poorest (Q1). In this case, rank-ordering each wealth quintile by their antibiotic use matched their wealth ordering.

Comparatively, when vaccines were distributed, inequities among antibiotic use by wealth quintile became insignificant (Fig. 2d). Antibiotic use for all wealth quintiles was nearly the same and approached 0% of infected individuals requiring antibiotics (on average, 1% [0.1–2] of infected children require antibiotics). This indicated reduced demand for antibiotics: vaccination causes reduced illness severity (Fig. 2b), so fewer individuals required antibiotics for treatment. There remained early peaks in antibiotic use; these spikes are in the early stages of the simulation when vaccination was still increasing and match the occurrence of seasonal outbreaks (Fig. 1b). Moreover, these increases in antibiotic usage were equally seen across all wealth quintiles, an equity improvement compared to when there were no vaccines and the poorest required the most doses of antibiotics and treatment.

**Fig. 3:**
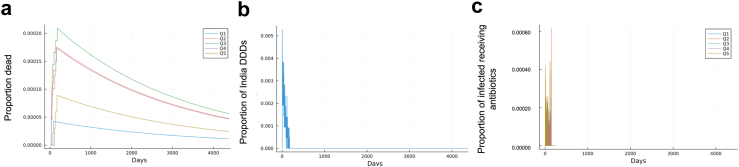
Increasing vaccines to near universal coverage (90%) has health equity benefits. Fully calibrated whole-India agent-based model run from 2016 to 2028 to determine health and antibiotic use impacts of increasing PCV13 and Hib-containing-pentavalent vaccination to near-universal levels. (a) Cumulative mortality curves by wealth quintile (total proportion of wealth quintile that has succumbed to *S. pneumoniae* or Hib) under a scenario of increased vaccination. (b) Proportion of total India daily defined doses (DDDs) of antibiotics used to treat *S. pneumoniae* and Hib for the same scenario as in (a). (c) Proportion of infected individuals receiving antibiotics by wealth quintile for the same scenario as in (a).

Near-universal vaccination would improve equity of health outcomes (Fig. 3a). Disease mortality is eclipsed by population growth at the same time for each wealth quintile (from day 208 for Q1 to 260 for Q3), indicating that increasing vaccinations has equal and equitable outcomes in reducing infections across all wealth quintiles. Antibiotic usage across wealth quintiles would show no statistical differences (Fig. 3b) and no excess use peaks as compared to when vaccination was at 2016 levels. All wealth quintiles have statistically similar stochastic trajectories (Fig. 3c), and no wealth quintile shows greater fluctuations than others, underscoring that increasing vaccination drives not only a decrease in antibiotics but equitable use of antibiotics and reduces the burden on the poorest individuals.

### Projected health impacts of near-universal vaccine coverage

We estimated additional reductions in infection and antibiotic use if vaccines were to be distributed near-universally (at 90% coverage). We simulated an increase in vaccinations from 2016 levels of DPT3 coverage (Supplementary Fig. S1) to 90% (i.e., near universal immunization coverage) across all of India over the same amount of time as it took to get to 2016 levels of coverage. To facilitate interpretation, we display disease incidence from modeled outbreaks of person-to-person spread, excluding the randomly introduced infections that we also model, to better visualize when outbreak-driven incidence reaches zero (Fig. 3a). We show the associated outbreak-driven antibiotic use from serotypes that are targeted by vaccination, defined as “excess” (Fig. 3b; Fig. 3c), meaning that we are excluding antibiotic use that would not be impacted by vaccination because the serotypes in question are not targeted by vaccines. Outbreak-driven disease incidence would drop to 0 after vaccination has increased by 11.3 percentage points [10.7–19.8]; this corresponds to a national average of 69% vaccine coverage. This transition is marked by the trend in Fig. 3a going from an increasing mortality trend to decreasing. Likewise, antibiotic use converged across wealth quintiles (Fig. 3b), and there are no excess use peaks as compared to when vaccination was at 2016 levels (Fig. 3c). Overall, we estimate that 90% vaccination would result in 71,535 [64,434–79,130] yearly deaths averted from *S. pneumoniae* and Hib.

**Fig. 4:**
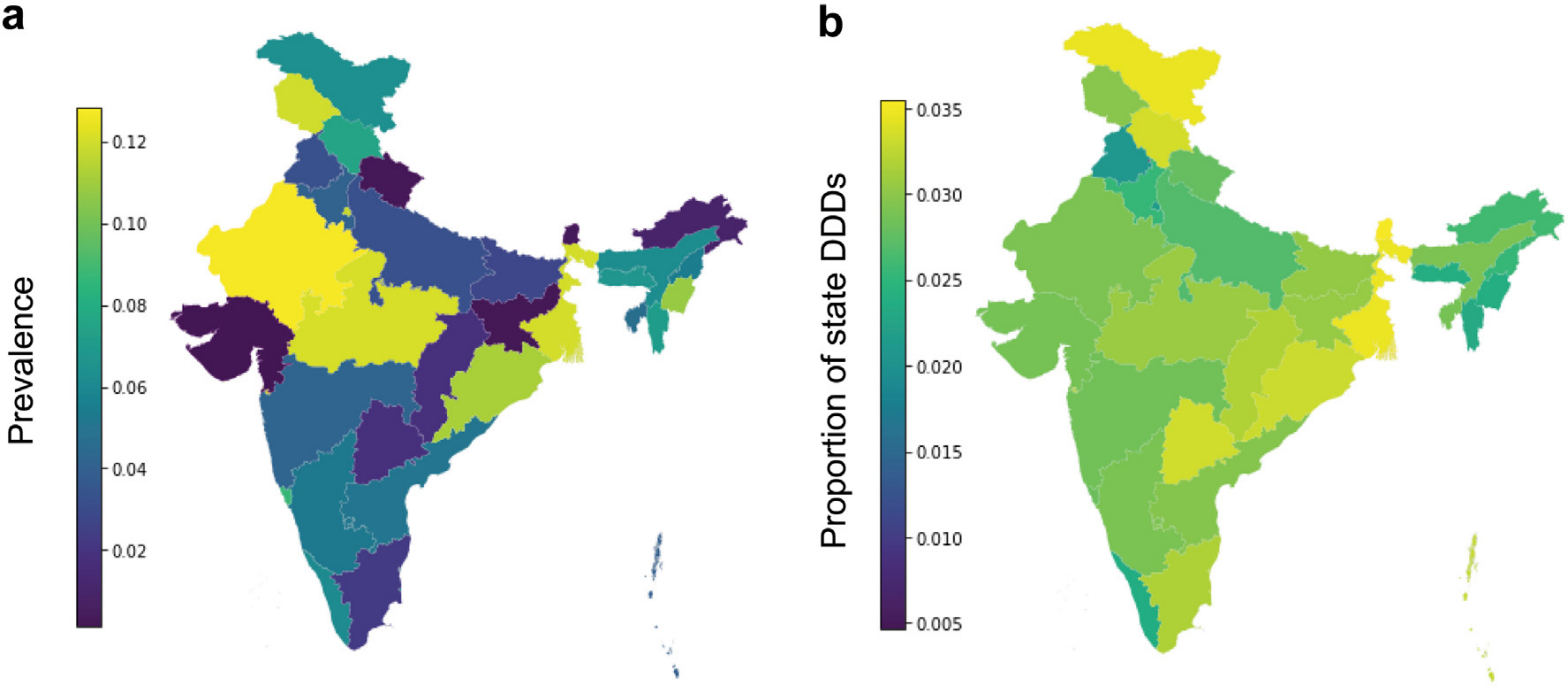
Geographic impacts of increasing vaccination to 90%. Fully calibrated whole-India agent-based model run from 2016 to 2028 to determine health and antibiotic use impacts of increasing PCV13 and Hib-containing-pentavalent vaccination to near-universal levels. (a) Steady-state disease prevalence from *S. pneumoniae* and Hib with 90% vaccination. (b) Reductions in antibiotic use from increasing vaccinations under steady-state prevalence and disease dynamics.

We geographically examined the impacts of increasing vaccination on steady-state disease prevalence and antibiotic use from *S. pneumoniae* and Hib (Fig. 4). As vaccine coverage was now equivalent across all states, our results reflect the impact of wealth quintile and the social network within each state on disease dynamics and antibiotic use. Disease prevalence varied widely across India and across neighboring states (Fig. 4a) while reductions in antibiotic use were less variable (Fig. 4b). We identified two regions that would have low steady-state disease prevalence: large portions of Western, Southern, and Central India and a band within mountainous Northern India extending to the Northeast corridor. The lowest steady state disease prevalence was found in Gujarat, 63.4% [42.8–68.9] less than the average across the rest of India. Gujarat is part of the Western, Southern, and Central India states with overall low prevalence that spans to Tamil Nadu. The band of Northern India with low prevalence spans from Punjab to Bihar and Jharkhand, omitting the National Capital Territory of Delhi.

We projected that Rajasthan would have the highest prevalence, 32.5% [28.7–39.4] greater than the rest of India. Prevalence would remain high in the states of Madhya Pradesh (11.2% [10.3–11.9]), Odisha (9.3% [9.1–9.5]), and West Bengal (12.0% [11.8–12.4]) and was lowest in Gujarat (1% [0.1–1.1]). Comparatively, reductions in antibiotic use were estimated to be more homogenous across all states. Reductions in antibiotic use would be lowest in Punjab (2.5% [2.2–2.8]) and greatest in Ladakh and Jammu and Kashmir (3.4% [3.3–3.5]). Regionally, Indian Ocean-bordering states across the west of India (Rajasthan, Gujarat, Maharashtra, and Karnataka) would have the greatest homogeneity in antibiotic use reduction (coefficient of variation of 3% [2–6]). Even across all of India, variation would remain minimal (coefficient of variation of 11% [9–12]), and there were minimal distinct geographical trends, underscoring the complex and challenging-to-predict nature of *S. pneumoniae* and Hib transmission across heterogeneous social networks and with socioeconomic variables that were modeled in this work.

## Discussion

Antibiotic consumption is growing rapidly in LMICs. From 2000 to 2015, per capita consumption of World Health Organization-designated Watch antibiotics (those which should be used sparingly because of AMR risk in the AwaRe classification) increased by 165% in LMICs.^50^ In 2019, India consumed an estimated 5071 million DDDs of antibiotic use leading to Southeast Asia alone accounting for 6.5% of global antibiotic usage; an estimated 54.9% of DDDs were from WHO Watch classified antibiotics.^51^^,^^52^ We developed an agent-based model to dynamically estimate the impact of childhood vaccinations on antibiotic use in India. Our model retrospectively considered the health and antibiotic impacts from 2004 to 2016 due to vaccines part of India's Universal Immunization Program and projected the future impacts to 2028 of increasing such vaccinations. Though we make certain simplifying assumptions and did not model explicit historical events in India, our findings align with available estimates of *S. pneumoniae* and Hib incidence and mortality in India. In particular, we estimate that in 2015, post initial vaccination efforts, yearly disease mortality from pneumonia was 8 per 1000 children (Fig. 2), in line with existing estimates of 9 per 1000 children.^24^ Likewise, in 2015, we estimate that incidence of symptomatic diseases was 35 cases per 1000 children (Fig. 1), in line with existing estimates for severe pneumonia of 31 cases per 1000 children.^53^ Finally, we identify Madhya Pradesh as having among the greatest prevalence of disease (Fig. 4a), consistent with previous work that has shown the state to have some of the highest disease burden across India.^24^^,^^53^ Together, this provides credibility in our model's future projections because it can recreate existing dynamics. Nonetheless, these are likely underestimates of the actual burden of *S. pneumoniae* and Hib burden because our model is itself likely an underestiamte of *S. pneumoniae* and Hib cases.

We estimated that PCV13 and Hib-containing-pentavelent vaccines implemented at the 2016 levels of DPT3 vaccination have substantially reduced antibiotic use attributable to these diseases (Fig. 1; by about 60%) and that continued vaccination would reduce the preventable antibiotic use for these diseases almost completely (Fig. 3). We projected that the benefits of having both vaccines distributed at 2016 DPT3 levels could be seen within 1–3 years of increasing vaccination rates. Our estimates for the health benefits of 90% immunization coverage are consistent with previous studies: we estimate that averted deaths would be as many as 71,000 per year (based on the population of India in 2021), in line with other modeling studies that have estimated that near-universal immunization coverage would avert 67,000 deaths per year.^54^ These estimates and the associated reductions in antibiotic use may be underestimates because (a) they are based on data that acknowledges it is an underestimate of the true burden of *S. pneumoniae* and Hib, and (b) we make conservative assumptions about antibiotic access, suggesting that averted antibiotic use from *S. pneumoniae* and Hib may be greater than estimated. However, total antibiotic use may not necessarily decrease if antibiotic use from other diseases increases, or there are challenges in having clinicians not prescribe antibiotics as readily for even minor symptoms resulting from *S. pneumoniae* and Hib. Nevertheless, our results underscore the potential for averted deaths and antibiotic use from increasing PCV13 and Hib-containing-pentavalent vaccination. These insights are particularly useful in low- and middle-income countries where antibiotic use is increasing, AMR represents a significant health burden, and access to second- or third-line antibiotics or alternative disease mitigation strategies such as increased sanitation is often challenging.

Our modeling underscores the need to ensure that the ensure that the poorest wealth quintile does not disproportionately face the burden of each disease: we show that with increasing vaccination to 2016 levels of coverage, inequities in mortality are reduced and inequities in overall antibiotic use are nearly eliminated. The wealthiest, who can obtain antibiotics easily, are likely to experience the greatest decrease in antibiotic use. High demand for antibiotics among wealthy individuals was driven by them experiencing respiratory disease symptoms and seeking treatment; on the other hand, although poorer individuals were also experiencing symptoms, they were unable to obtain antibiotics. A detailed examination of the equity implications of our work are available in Supplementary Appendix 2.

By projecting the impact of future vaccination campaigns, we provided new insight into the nonlinear dynamics between vaccination, health burden, and antibiotic use. This approach more accurately reflects the process of vaccine distribution that occurs in practice and is critical to informing policy. However, future work is required to better probe causality between rates of vaccination and the prevalence of various bacterial serotypes and antibiotic resistance of those associated pathogens. This work did not explicitly model transmission by particular serotypes or the serotype replacement effect for *S. pneumoniae*; rather, the bulk behavior of all serotypes were modeled with individual-level variability that reflected the variance across serotypes. However, we are unable to extract antibiotic use related to specific serotypes or explicitly model serotype dynamics, which could be valuable for future work. For instance, an explicit model with type of vaccine, individual disease serotypes, and antibiotic classes could enable understanding which serotypes are causing the most antibiotic use by antibiotic class and test strategies to avert such use and the potential impacts on antimicrobial resistance. Likewise, our modeling indicates the complex and nonlinear nature of how disease dynamics, vaccination, antibiotic use, and wealth quintile interact: future work includes using dynamical modeling analyses including perturbation tests and linearization processes to better understand this system and develop guiding principles and heuristics to advise the impacts of vaccination policy on antibiotic use and health equity.

Our approach has many caveats. Firstly, we only modeled the transmission of *S. pneumoniae* and Hib—we did not account for the additional impacts of other diseases and their spread on hospital utilization or how hospital visits may impact underlying transmission and antibiotic use. Similarly, we did not have the data to explicitly model how particular symptoms—such as otitis media for *S. pneumoniae* or an infection being an inpatient versus outpatient case—causally drive antibiotic use. Instead, the impact of various disease manifestations on antibiotic use was modeled implicitly through heterogeneity in the probability of antibiotic usage and the impact it may have had on vaccination. Nevertheless, this approach makes it challenging to directly extract the antibiotic use attributable to individuals with particular symptoms or clinical characteristics of their infections. Further data could also help refine our modeling assumptions: in some limited cases, the most detailed data we could find was from countries outside India. Although we included uncertainty in the underlying source data when integrating it into our model and our calibration procedure enables robustness to underlying source data, further data that details the epidemiological state of *S. pneumoniae* and Hib in India in particular would be valuable for advancing modeling efforts. Likewise, detailed data on antibiotic usage by wealth quintile, the characteristics of those seeking antibiotics, and the type of antibiotics they seek would be valuable for more precisely informing antibiotic use in our model. Additional data on *S. pneumoniae* and Hib burden separately and a more mechanistic model would also be valuable for providing insights into the impact of vaccination on each individual disease, and this is an area of future work. Finally, we modeled the vaccination campaigns of India's Universal Immunization Programme as a linear increase in vaccination coverage from zero to current levels of coverage when modeling retrospective vaccinations and then a linear increase from current levels to near-universal coverage when modeling future vaccinations. However, actual implementation of vaccines was likely not linear, and source data on the distribution of vaccines would be valuable for improving modeling efforts.

Although we were modeling an extended period of time (2004–2028), we are assuming that state-level variables such as the distribution of people by wealth quintile within a particular district stay constant. Similarly, future work includes determining the impact of varying assumptions on the underlying demographic model to 2028 on averted burden and antibiotic use. At the time of writing, the full set of data needed to parameterize this model was only available pre-COVID, but as post-COVID data becomes available, future work also includes using updated data to project the health and antibiotic use impacts of *S. pneumoniae* and Hib vaccination post-COVID. This remains a critical area of work as public health policy sets post-COVID vaccination priorities. Beyond that, while we were attempting to model human behavior relevant for disease spread, we are not accounting for differential social norms and other variables that may drive clustered outbreaks.^55^, ^56^, ^57^ Previous work has indicated the importance of considering these dynamics, and they may impact the development of the best strategies for outbreak management and antibiotic stewardship and reducing AMR.

AMR is growing fastest in LMICs.^58^ Estimates suggest that AMR could lead to an annual loss in GDP of 5–7% by 2050 in developing countries^59^ and an annual mortality burden of 8.8 million in the Asia and Africa regions.^60^ Reduction in antibiotic consumption may result in decreases in antibiotic resistance not only at the individual level, but also at the community, country, and regional level.^61^ Our work provides the first-ever dynamic analysis of how vaccination reduces antibiotic use at a subnational scale and by wealth quintile and how further increasing vaccinations may improve health equity. We demonstrate key features of the impact of future vaccinations and indicate that significant progress to health impacts and health equity can be made that motivate the widespread distribution of PCV and Hib-containing-pentavalent vaccination and additional benefits that may arise if these vaccines are made available at near universal levels in India.

## Contributors

RL, AN and EK conceived the study. CKK, GGP, and AS did the literature search. CKK and ACG did the data analyses and modeling. CKK wrote the initial draft. All authors had access to the data and ACG verified the data. All authors provided scientific input, edited, and approved the final draft.

## Data sharing statement

All code and data used are available online: https://github.com/Agleason1/Agent-Based-Infection-Model--S.-Pneumoniae.

## Editor note

The Lancet Group takes a neutral position with respect to territorial claims in published maps and institutional affiliations.

## Declaration of interests

The authors declare no competing interests.

## References

[1] Anderson R.M. (1992). The concept of herd immunity and the design of community-based immunization programmes. Vaccine.

[2] Li X., Mukandavire C., Cucunubá Z.M. (2021). Estimating the health impact of vaccination against ten pathogens in 98 low-income and middle-income countries from 2000 to 2030: a modelling study. Lancet.

[3] Okeke I.N., de Kraker M.E., Van Boeckel T.P. (2024). The scope of the antimicrobial resistance challenge. Lancet.

[4] Birger R., Antillón M., Bilcke J. (2022). Estimating the effect of vaccination on antimicrobial-resistant typhoid fever in 73 countries supported by Gavi: a mathematical modelling study. Lancet Infect Dis.

[5] Kumar C.K., Sands K., Walsh T.R. (2023). Global, regional, and national estimates of the impact of a maternal Klebsiella pneumoniae vaccine: a Bayesian modeling analysis. PLoS Med.

[6] Fu H., Lewnard J.A., Frost I., Laxminarayan R., Arinaminpathy N. (2021). Modelling the global burden of drug-resistant tuberculosis avertable by a post-exposure vaccine. Nat Commun.

[7] Bloom D.E., Black S., Salisbury D., Rappuoli R. (2018). Antimicrobial resistance and the role of vaccines. Proc Natl Acad Sci U S A.

[8] Micoli F., Bagnoli F., Rappuoli R., Serruto D. (2021). The role of vaccines in combatting antimicrobial resistance. Nat Rev Microbiol.

[9] Buckley B.S., Henschke N., Bergman H. (2019). Impact of vaccination on antibiotic usage: a systematic review and meta-analysis. Clin Microbiol Infect.

[10] Schueller E., Nandi A., Joshi J., Laxminarayan R., Klein E.Y. (2021). Associations between private vaccine and antimicrobial consumption across Indian states, 2009–2017. Ann N Y Acad Sci.

[11] Klein E.Y., Schueller E., Tseng K.K., Morgan D.J., Laxminarayan R., Nandi A. (2020).

[12] Walsh T.R., Weeks J., Livermore D.M., Toleman M.A. (2011). Dissemination of NDM-1 positive bacteria in the New Delhi environment and its implications for human health: an environmental point prevalence study. Lancet Infect Dis.

[13] Goyal P.K., Semwal A., Prakash A., Medhi B. (2019). Emerging antimicrobial resistance and newer tools to address the resistance. Indian J Pharmacol.

[14] Santhosh K.S., Deekshit V.K., Venugopal M.N., Karunasagar I., Karunasagar I. (2017). Multiple antimicrobial resistance and novel point mutation in fluoroquinolone-resistant Escherichia coli isolates from Mangalore, India. Microb Drug Resist.

[15] Marathe N.P., Berglund F., Razavi M. (2019). Sewage effluent from an Indian hospital harbors novel carbapenemases and integron-borne antibiotic resistance genes. Microbiome.

[16] Lewnard J.A., Lo N.C., Arinaminpathy N., Frost I., Laxminarayan R. (2020). Childhood vaccines and antibiotic use in low- and middle-income countries. Nature.

[17] Klugman K.P., Black S. (2018). Impact of existing vaccines in reducing antibiotic resistance: primary and secondary effects. Proc Natl Acad Sci U S A.

[18] King L.M., Andrejko K.L., Kabbani S. (2024). Outpatient visits and antibiotic use due to higher-valency pneumococcal vaccine serotypes. J Infect Dis.

[19] Allwell-Brown G., Hussain-Alkhateeb L., Sewe M.O. (2021). Determinants of trends in reported antibiotic use among sick children under five years of age across low-income and middle-income countries in 2005–17: a systematic analysis of user characteristics based on 132 national surveys from 73 countries. Int J Infect Dis.

[20] McGurn A., Watchmaker B., Adam K. (2021). Socioeconomic status and determinants of pediatric antibiotic use. Clinical pediatrics.

[21] Dorta H.G., Nandi A. (2023). Patterns of antibiotic use for acute respiratory infections in under-three-year-old children in India: a cross-sectional study. J Glob Health.

[22] Smith J., Lipsitch M., Almond J.W. (2011). Vaccine production, distribution, access, and uptake. Lancet.

[23] Bhadoria A.S., Mishra S., Singh M., Kishore S. (2019). National immunization programme–mission Indradhanush programme: newer approaches and interventions. Indian J Pediatr.

[24] Wahl B., Sharan A., Knoll M.D. (2019). National, regional, and state-level burden of Streptococcus pneumoniae and Haemophilus influenzae type b disease in children in India: modelled estimates for 2000–15. Lancet Global Health.

[25] Vashishtha V.M., Kumar P. (2013). 50 years of immunization in India: progress and future. Indian Pediatr.

[26] Summan A., Nandi A., Deo S., Laxminarayan R. (2021). Improving vaccination coverage and timeliness through periodic intensification of routine immunization: evidence from Mission Indradhanush. Ann N Y Acad Sci.

[27] Schueller E., Nandi A., Summan A. (2022). Public finance of universal routine childhood immunization in India: district-level cost estimates. Health Policy Plan.

[28] Summan A., Nandi A., Shet A., Laxminarayan R. (2023). The effect of the COVID-19 pandemic on routine childhood immunization coverage and timeliness in India: retrospective analysis of the National Family Health Survey of 2019–2021 data. Lancet Reg Health Southeast Asia.

[29] Megiddo I., Colson A.R., Nandi A. (2014). Analysis of the universal immunization programme and introduction of a rotavirus vaccine in India with IndiaSim. Vaccine.

[30] Megiddo I., Colson A., Chisholm D., Dua T., Nandi A., Laxminarayan R. (2016). Health and economic benefits of public financing of epilepsy treatment in India: an agent-based simulation model. Epilepsia.

[31] Nandi A., Colson A.R., Verma A., Megiddo I., Ashok A., Laxminarayan R. (2016). Health and economic benefits of scaling up a home-based neonatal care package in rural India: a modelling analysis. Health Policy Plan.

[32] Megiddo I., Klein E., Laxminarayan R. (2018). Potential impact of introducing the pneumococcal conjugate vaccine into national immunisation programmes: an economic-epidemiological analysis using data from India. BMJ Glob Health.

[33] Nandi A., Megiddo I., Ashok A., Verma A., Laxminarayan R. (2017). Reduced burden of childhood diarrheal diseases through increased access to water and sanitation in India: a modeling analysis. Soc Sci Med.

[34] Gleason A., Kumar C.K., Klein E., Laxminarayan R., Nandi A. (2024). Effect of rotavirus vaccination on the burden of rotavirus disease and associated antibiotic use in India: a dynamic agent-based simulation analysis. Vaccine.

[35] Dhirar N., Dudeja S., Khandekar J., Bachani D. (2018). Childhood morbidity and mortality in India—analysis of national family health survey 4 (NFHS-4) findings. Indian Pediatr.

[36] Summan A., Nandi A., Laxminarayan R. (2022).

[37] Mistry D., Litvinova M., Chinazzi M. (2020). Inferring high-resolution human mixing patterns for disease modeling. arXiv.

[38] Hoertel N., Blachier M., Blanco C. (2020). A stochastic agent-based model of the SARS-CoV-2 epidemic in France. Nat Med.

[39] Moghadas S.M., Fitzpatrick M.C., Sah P. (2020). The implications of silent transmission for the control of COVID-19 outbreaks. Proc Natl Acad Sci U S A.

[40] Kumar C.K., Balasubramanian R., Ongarello S., Carmona S., Laxminarayan R. (2022). SARS-CoV-2 testing strategies for outbreak mitigation in vaccinated populations. PLoS One.

[41] Lloyd-Smith J.O., Schreiber S.J., Kopp P.E., Getz W.M. (2005). Superspreading and the effect of individual variation on disease emergence. Nature.

[42] Tsang T.K., Wang C., Fang V.J. (2023). Reconstructing household transmission dynamics to estimate the infectiousness of asymptomatic influenza virus infections. Proc Natl Acad Sci U S A.

[43] Cobey S., Lipsitch M. (2012). Niche and neutral effects of acquired immunity permit coexistence of pneumococcal serotypes. Science.

[44] Domenech de Cellès M., Arduin H., Lévy-Bruhl D. (2019). Unraveling the seasonal epidemiology of pneumococcus. Proc Natl Acad Sci U S A.

[45] Tristram S., Jacobs M.R., Appelbaum P.C. (2007). Antimicrobial resistance in Haemophilus influenzae. Clin Microbiol Rev.

[46] Hawkins P.A., Akpaka P.E., Nurse-Lucas M. (2017). Antimicrobial resistance determinants and susceptibility profiles of pneumococcal isolates recovered in Trinidad and Tobago. J Glob Antimicrob Resist.

[47] AlonsoDeVelasco E., Verheul A., Verhoef J., Snippe H. (1995). Streptococcus pneumoniae: virulence factors, pathogenesis, and vaccines. Microbiol Rev.

[48] Jaiswal N., Singh M., Thumburu K.K. (2014). Burden of invasive pneumococcal disease in children aged 1 month to 12 years living in South Asia: a systematic review. PLoS One.

[49] Steinhoff M.C. (1998). Invasive Haemophilus influenzae disease in India: a preliminary report of prospective multihospital surveillance. IBIS (Invasive Bacterial Infections Surveillance) Group. Pediatr Infect Dis J.

[50] Klein E.Y., Milkowska-Shibata M., Tseng K.K. (2021). Assessment of WHO antibiotic consumption and access targets in 76 countries, 2000–15: an analysis of pharmaceutical sales data. Lancet Infect Dis.

[51] Browne A.J., Chipeta M.G., Haines-Woodhouse G. (2021). Global antibiotic consumption and usage in humans, 2000–18: a spatial modelling study. Lancet Planet Health.

[52] Koya S.F., Ganesh S., Selvaraj S., Wirtz V.J., Galea S., Rockers P.C. (2022). Consumption of systemic antibiotics in India in 2019. Lancet Reg Health Southeast Asia.

[53] Farooqui H., Jit M., Heymann D.L., Zodpey S. (2015). Burden of severe pneumonia, pneumococcal pneumonia and pneumonia deaths in Indian states: modelling based estimates. PLoS One.

[54] Constenla D., Liu T. (2019). Estimating the economic impact of pneumococcal conjugate, Haemophilus influenzae type b and rotavirus vaccines in India: national and state-level analyses. Vaccine.

[55] Betsch C., Korn L., Sprengholz P. (2020). Social and behavioral consequences of mask policies during the COVID-19 pandemic. Proc Natl Acad Sci U S A.

[56] Hruschka D.J., Brewis A.A., Wutich A., Morin B. (2011). Shared norms and their explanation for the social clustering of obesity. Am J Public health.

[57] Reid A.E., Cialdini R.B., Aiken L.S. (2010). Handbook of behavioral medicine.

[58] O'Neill J. (2014).

[59] Dadgostar P. (2019). Antimicrobial resistance: implications and costs. Infect Drug Resist.

[60] Murray C.J., Ikuta K.S., Sharara F. (2022). Global burden of bacterial antimicrobial resistance in 2019: a systematic analysis. Lancet.

[61] Allel K., Day L., Hamilton A. (2023). Global antimicrobial-resistance drivers: an ecological country-level study at the human–animal interface. Lancet Planet Health.

