# Supplementary material for: Routine immunization against Streptococcus pneumoniae and Haemophilus influenzae type B and antibiotic consumption in India: a dynamic modeling analysis
Source: Lancet Reg Health Southeast Asia. 2024 Oct 16;31:100498. doi: 10.1016/j.lansea.2024.100498 (PMC11530913; doi:10.1016/j.lansea.2024.100498)
Supplement: Supplementary Appendix [file mmc1.docx]

**Equity impacts of near-universal vaccine coverage**

Equal demand for antibiotics indicates that it is irrelevant that the wealthiest may have greater access to antibiotics because total demand for antibiotics from symptom-presenting individuals remains below the point at which the wealth quintiles differentiate; in turn, this underscores that there are not poorer individuals who are going without treatment as before. Increasing vaccination to near-universal levels underscores these patterns: antibiotic use decreases monotonically and surges in demand are removed. Moreover, total mortality from *S. pneumoniae* and Hib decreases and becomes statistically indistinguishable across wealth quintiles, even between the poorest and wealthiest. However, we modeled the two vaccines as increasing in coverage concurrently because of the absence of data on PCV vaccine coverage by district; in reality, the two vaccines were not distributed concurrently with Hib vaccination becoming part of UIP earlier than PCV vaccination.^1^ We encourage that vaccination data at the district level for all vaccines be made available as it will aid future analyses and policymaking. Moreover, in light of the COVID-19 pandemic, universal immunization program vaccination rates have dropped, meaning that additional effort will be required to get vaccination back to the levels of coverage aimed for by Mission Indradhanush.^2^ Our results underscore the importance of increasing such vaccinations on not only children’s health but equity. Nevertheless, we acknowledge that our future projects cannot accurately reflect the subpopulations that have been most impacted by COVID-19 (both in terms of direct effects from COVID-19 and indirect effects in terms of COVID-19 causing a reduction in their universal immunization program vaccination rates). These effects are not yet fully known, and future work will consider incorporating such inequities as they are identified. Nevertheless, it is likely that our estimates of vaccination and averted antibiotic use in 2016 represent the current state as additional gains in vaccination from 2016 to 2019 were largely erased by the COVID-19 pandemic.^2^

Highlighting the complex relationship between vaccination, disease dynamics, and antibiotic use, we find that the poorest do not necessarily have the worst outcomes as there is a non-monotonic ordering of wealth quintiles by infection and antibiotic outcomes. Moreover, wealth quintile ordering changes throughout the simulation, reflecting the importance of dynamic models in evaluating health and equity impacts of vaccination. Q3 consistently has the worst health outcomes: we posit that this is because antibiotics are generally most readily available to the wealthiest while vaccines are more universally available and often emphasize accessing the poorest, meaning that a middle class has the least access to both mitigation measures. Consistent with this hypothesis, we note that Q1 and Q5 consistently have the best health outcomes when vaccines are present. The rank-ordering of wealth quintiles by health outcomes may change over time because of the supply of susceptible individuals: while wealthier individuals are less likely to become sick, they remain susceptible. Thus, once the disease has progressed through other groups and conveyed immunity to such individuals, only wealthier individuals will be left as susceptible. Because both *S. pneumoniae* and Hib spread through asymptomatic carriers, it is likely that such individuals will eventually become infected. When vaccines are present, the non-monotonic nature of ordering wealth quintiles by outcomes reflects the complex nature of vaccines and antibiotics being available differently for each wealth quintile compounded with susceptible supply and localized herd immunity. Finally, while infection seems to increase in the long term even with vaccinations, this is due to waning immunity of vaccines (Fig. 1B), and the infected children are older; therefore, their infection severity is less, so these infections present a lesser burden. Future work emphasizes better understanding these patterns.

**References**

1 Bloom DE, Cadarette D, Ferranna M, Nandi A, Shet A. Value of Vaccination in India: Past, Present, and Future Prospects. .

2 Summan A, Nandi A, Shet A, Laxminarayan R. The effect of the COVID-19 pandemic on routine childhood immunization coverage and timeliness in India: retrospective analysis of the National Family Health Survey of 2019–2021 data. *The Lancet Regional Health-Southeast Asia* 2023; **8**.
