# Supplementary material for: Routine immunization against Streptococcus pneumoniae and Haemophilus influenzae type B and antibiotic consumption in India: a dynamic modeling analysis
Source: Lancet Reg Health Southeast Asia. 2024 Oct 16;31:100498. doi: 10.1016/j.lansea.2024.100498 (PMC11530913; doi:10.1016/j.lansea.2024.100498)
Supplement: Supplemental Table [file mmc2.docx]

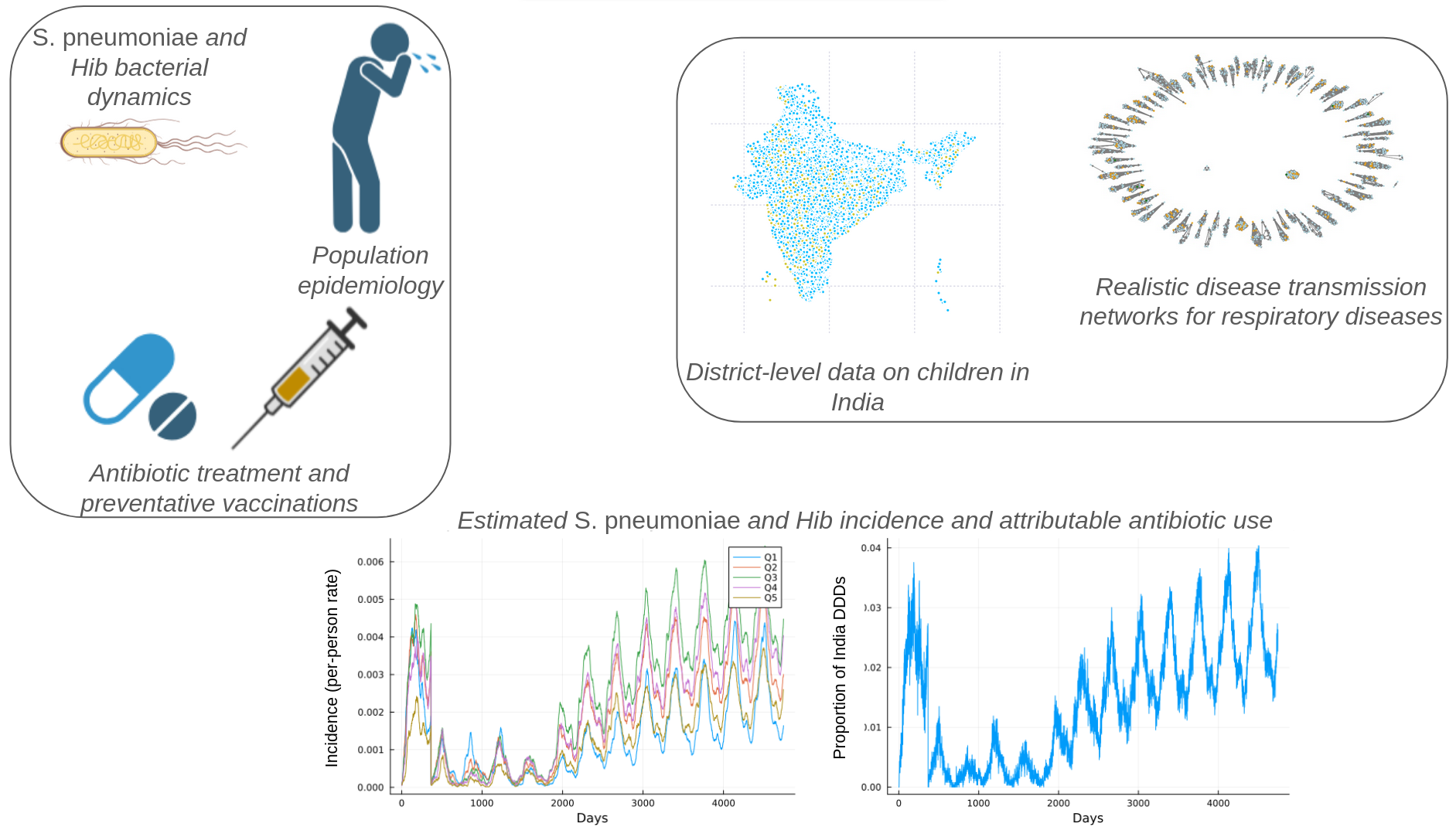
Supplementary Figure 1. Schematic diagram of different model inputs (boxed) and the desired model outputs for analysis.

Appendix Table 1. Complete list of model parameters.

| **Parameters** | **Value** | | **Source** |
| --- | --- | --- | --- |
| I. **District attributes (values vary by district/state)** | | | |
| Growth rate |  | | NFHS-4 |
| Population size |  | | NFHS-4, NFHS-5 (approximated for all years using growth rate and 2017 size) |
| Age distribution |  | | NFHS-4 (approximated using growth rate) |
| Wealth quintile distribution |  | | NFHS-5 |
| Proportion vaccinated |  | | NFHS-5 |
| Incidence of pneumonia-like disease in India |  | | Farooqui et al., 2015^1^ (used the same incidence values for all ages <14) |
| **II. Basic individual characteristics** | | | |
| Age | Sampled from associated district’s age distribution | |  |
| Wealth quintile | Sampled from associated district’s wealth quintile distribution | |  |
| Vaccination status | Probability based on proportion vaccinated in associated district | |  |
| Vaccine efficacy (if vaccinated) | [0.63-0.89] | | van der Linden, 2016^2^ (for *S. pneumoniae*); Morris et al., 2008^3^ (for Hib) |
| **III. Disease dynamics and infected characteristics** | | | |
| Basic reproductive number | Strain-dependent: see citation | | Lipsitch, 1997^4^ |
| Incubation period (days) | [1-3] | | Cobey and Lipsitch^5^ |
| Peak transmission (days after incubation period) | [1-5] | |  |
| Duration infectious period (non-carrier) | [9-39] | |  |
| **Probability of carriage** | | | |
|  | *S. pneumoniae* | Hib |  |
| 0 to <1 years of age | 0.152 | 0.112 | *S. pneumoniae* data come from Kumar et al., 2021^6^ and Hib data come from Sekhar et al., 2009^7^ and Jain et al., 2005^8^ – these data are used as the initial conditions in the model for the calibration period, not the absolute values in the model. |
| 1 to <2 years of age | 0.019 | 0.112 |  |
| 2 to 5 years of age | 0.004 | 0.085 |  |
| 5 to 10 years of age | 0 | 0.0785 |  |
| 10 to <14 years of age | 0 | 0.059 | Inferred from age trends in above citations. |
| Carrier clearance rate (per month) | [0.64-0.75] | | Leino et al., 2009^9^ |
| **Infection mortality by serotype (if untreated)** | | | |
| <5 years | 0.0425 | | Inverarity et al., 2011^10^ |
| 5+ years | 0.0413 | |  |
| **Reinfection Probability by Serotype** | | | |
| Past infection by the same serotype | 0 | | Inverarity et al., 2011^10^ |
| New serotype (conserved variant, infected by a conserved variant in the past) | 0.05 | |  |
| New serotype (conserved variant, not infected by a conserved variant in the past) | 0.8 | |  |
| New serotype (not conserved variant) | 0.8 | |  |
| **Probability to seek antibiotics (seek until cured, or until each antibiotic variant has been tried once)** | | | |
| Wealth quintile 1 | 0.2 | | Expert assumptions based on underlying trends reported in Dorta et al., 2023^11^ |
| Wealth quintile 2 | 0.6 | |  |
| Wealth quintile 3 | 0.8 | |  |
| Wealth quintile 4 | 0.95 | |  |
| Wealth quintile 5 | 1 | |  |
| **Days until an antibiotic is received (if seeking antibiotics)** | | | |
| First antibiotic | [1-5] | | Expert assumptions |
| Second or third antibiotic | [4-7] | |  |
| **Probability of serotype resistance** | | | |
| Penicillin resistant | 0.0108 | | Manoharan et al., 2017^12^ (scaled to exclude intermediate resistance) |
| Erythromycin resistant | 0.3737 | |  |
| Chloramphenicol resistant | 0.09 | |  |
| **IV. Vaccinated characteristics** | | | |
| Transmission by day relative to unvaccinated | 0.75 | | Assumption; sensitivity tested in Appendix Table 2. |
| Infection mortality relative to unvaccinated, assuming that vaccine is ineffective in stopping symptoms | 0.75 | | Assumption |
| **Vaccine effectiveness against seeking antibiotics** | | | |
| 0 to <2 years of age | 0.062 | | Lewnard et al., 2020^13^ |
| 2 to <5 years of age | 0.2375 | |  |
| 5+ years of age | 0.2375 | | Extrapolation from Lewnard et al., 2020^13^ |


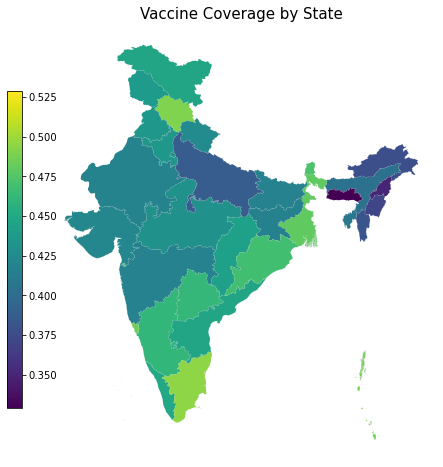
Supplementary Figure 2. DPT-3 vaccination coverage by state in India as of 2016.

| Assumption | | Antibiotic use from *S. pneumoniae* or Hib is 20% less than in main analysis | Vaccine efficacy against transmission is 10% less than in main analysis | Q5 and Q4 have 25% greater access to antibiotics than in main analysis |
| --- | --- | --- | --- | --- |
| Reduction in antibiotic use between 2016 DPT3 vaccine coverage and no vaccine coverage | | 48.3% [32.1 – 58.4] | 39.4% [30.3 – 49.7] | 62.5% [53.7 – 68.7] |
| Decrease in pneumoniae-like illness from PCV13 and DPT-Hib vaccination at 2016 DPT3 levels | | 36.8% [22.3 – 67.9] | 31.7 [19.6 – 56.9] | 36.8% [22.3 – 67.9] |
| Vaccination coverage increase required to make inequities in antibiotic use negligible | | 11.3 percentage points [10.7 – 19.8] | 19.4 percentage points [15.3 – 32.5] | 16.4 [11.2 – 24.5] |
| Total India DDDs attributable to *S. pneumoniae* and Hib antibiotic use | | 3.5% [3.1 – 4.6] | 5.4% [4.8 – 5.9] | 4.5 [3.7 – 4.7] |
| Expected percentage of patients taking antibiotics with no vaccines | | 2.0% [1.1 – 2.1] | 1.8% [0.9 – 1.9] | 1.9% [1.0 – 2.0] |
| Decrease in antibiotic usage from 2004 to 2016 as vaccination coverage reached DPT3 levels | Q1 | 28.5 [24.3 – 29.6] | 31.4 [29.7 – 41.0] | 33.6 [31.5 – 42.8] |
|  | Q2 | 46.3 [41.6 – 49.2] | 49.6 [43.4 – 52.1] | 52.4 [49.8 – 59.4] |
|  | Q3 | 65.3 [55.6 – 79.3] | 69.3 [57.8 – 74.3] | 72.1 [68.4 – 75.3] |
|  | Q4 | 75.5 [68.3 – 78.3] | 78.4 [75.4 – 91.2] | 78.4 [73.2 – 82.3] |
|  | Q5 | 82.1 [75.9 – 91.5] | 86.7 [82.1 – 94.5] | 82.6 [79.1 – 95.3] |

Appendix Table 2. Main results from sensitivity analyses of assumed parameters.

1. Farooqui H, Jit M, Heymann DL, et al. Burden of Severe Pneumonia, Pneumococcal Pneumonia and Pneumonia Deaths in Indian States: Modelling Based Estimates. *PLOS ONE* 2015;10(6):e0129191. doi: 10.1371/journal.pone.0129191

2. van der Linden M, Falkenhorst G, Perniciaro S, et al. Effectiveness of Pneumococcal Conjugate Vaccines (PCV7 and PCV13) against Invasive Pneumococcal Disease among Children under Two Years of Age in Germany. *PLoS One* 2016;11(8):e0161257. doi: 10.1371/journal.pone.0161257 [published Online First: 20160815]

3. Morris, S. K., Moss, W. J., & Halsey, N.. Haemophilus influenzae type b conjugate vaccine use and effectiveness. *The Lancet infectious diseases*, 2008;*8*(7), 435-443. doi: 10.1016/S1473-3099(08)70152-X

4. Lipsitch, M. Vaccination against colonizing bacteria with multiple serotypes. *Proceedings of the National Academy of Sciences*. 1997; 94(12):6571-6576

5. Cobey S, Lipsitch M. Niche and neutral effects of acquired immunity permit coexistence of pneumococcal serotypes. *Science*. 2012;335(6074):1376–80.

6. Kumar S, Purakayastha DR, Kapil A, et al. Carriage rates and antimicrobial sensitivity of pneumococci in the upper respiratory tract of children less than ten years old, in a north Indian rural community. *PLOS ONE* 2021;16(2):e0246522. doi: 10.1371/journal.pone.0246522

7. Sekhar S, Chakraborti A, Kumar R. Haemophilius influenzae colonization and its risk factors in children aged< 2 years in northern India. *Epidemiology & Infection* 2009; *137*(2), 156-160. doi: https://doi.org/10.1017/S095026880800071X .

8. Jain A, Kumar P, Awasthi S. High nasopharyngeal carriage of drug resistant Streptococcus pneumoniae and Haemophilus influenzae in North Indian schoolchildren. *Tropical Medicine & International Health*, 2005; *10*(3), 234-239. doi: 10.1111/j.1365-3156.2004.01379.x.

9. Leino T, Hoti F, Syrjänen R, et al. Clustering of serotypes in a longitudinal study of Streptococcus pneumoniae carriage in three day care centres. *BMC Infectious Diseases* 2008;8(1):173. doi: 10.1186/1471-2334-8-173

10. Inverarity D, Lamb K, Diggle M, et al. Death or survival from invasive pneumococcal disease in Scotland: associations with serogroups and multilocus sequence types. *J Med Microbiol* 2011;60(Pt 6):793-802. doi: 10.1099/jmm.0.028803-0 [published Online First: 20110310]

11. Dorta HG, & Nandi A. Patterns of antibiotic use for acute respiratory infections in under-three-year-old children in India: A cross-sectional study. *Journal of Global Health*, 2023; *13*. doi: 10.7189/jogh.13.04159.

12. Manoharan A, Manchanda V, Balasubramanian S, et al. Invasive pneumococcal disease in children aged younger than 5 years in India: a surveillance study. *The Lancet Infectious Diseases* 2017;17(3):305-12. doi: 10.1016/S1473-3099(16)30466-2

13. Lewnard JA, Lo NC, Arinaminpathy N, et al. Childhood vaccines and antibiotic use in low- and middle-income countries. *Nature* 2020;581(7806):94-99. doi: 10.1038/s41586-020-2238-4
